# Circulating tumor DNA: a noninvasive biomarker for tracking ovarian cancer

**DOI:** 10.1186/s12958-021-00860-8

**Published:** 2021-12-03

**Authors:** Fang Yang, Jun Tang, Zihao Zhao, Chunling Zhao, Yuancai Xiang

**Affiliations:** 1 Department of Physiology, Basic Medical College, Southwest Medical University, Luzhou, Sichuan Province China; 2 Department of Biochemistry and Molecular Biology, Basic Medical College, Southwest Medical University, Luzhou, Sichuan Province China

**Keywords:** Ovarian cancer, circulating tumor DNA, biomarker, cancer early detection, liquid biopsy

## Abstract

Ovarian cancer is the fifth leading cause of cancer-related mortality in women worldwide. Despite the development of technologies over decades to improve the diagnosis and treatment of patients with ovarian cancer, the survival rate remains dismal, mainly because most patients are diagnosed at a late stage. Traditional treatment methods and biomarkers such as cancer antigen-125 as a cancer screening tool lack specificity and cannot offer personalized combinatorial therapy schemes. Circulating tumor DNA (ctDNA) is a promising biomarker for ovarian cancer and can be detected using a noninvasive liquid biopsy. A wide variety of ctDNA applications are being elucidated in multiple studies for tracking ovarian carcinoma during diagnostic and prognostic evaluations of patients and are being integrated into clinical trials to evaluate the disease. Furthermore, ctDNA analysis may be used in combination with multiple “omic” techniques to analyze proteins, epigenetics, RNA, nucleosomes, exosomes, and associated immune markers to promote early detection. However, several technical and biological hurdles impede the application of ctDNA analysis. Certain intrinsic features of ctDNA that may enhance its utility as a biomarker are problematic for its detection, including ctDNA lengths, copy number variations, and methylation. Before the development of ctDNA assays for integration in the clinic, such issues are required to be resolved since these assays have substantial potential as a test for cancer screening. This review focuses on studies concerning the potential clinical applications of ctDNA in ovarian cancer diagnosis and discusses our perspective on the clinical research aimed to treat this daunting form of cancer.

## Introduction

Ovarian cancer is the fifth leading overall cause of cancer-related mortality in women worldwide and the second most common cause of gynecologic cancer-related deaths. Up to 95% of all ovarian malignancies are diagnosed as epithelial ovarian cancer (EOC) [1]. The most common subtype of EOC is the high-grade serous ovarian cancer (HGSOC) with a prevalence of 52%, followed by endometrioid (10%), mucinous (6%), and clear cell adenocarcinomas (6%) [2]. Various efforts have been aimed at treating ovarian carcinoma over the last 30 years, and the disease is curable at an early stage in 90% of patients [3]. Yet overall disease control remains poor, in part due to the resistance to chemotherapy or other targeted drugs [4–6], which are associated with multiple factors including genome wide mutations [7], epigenetic changes [8], dysfunctionality of DNA repair pathways [9], drug inactivation [10], particular platinum resistance of cancer stem cells (CSCs) [11], and tumor microenvironment [12]. The poor response to this recurrent disease is owing in part to the paucity of effective screening options to discover the specific or typical symptoms at an early stage. Therefore, there is currently an urgent need for optimizing biomarkers that can serve as diagnostic, prognostic, and potential targets for novel therapies that can be used to guide further testing, initiate treatment, and direct the choice of ovarian cancer treatment.

Researchers are searching for the dynamic biomarkers applied in non-invasive methods that can indicate the cancer characteristics, such as HE4 [13], transferrin receptor 1 (TFR1) [14], and cancer antigen 125 (CA-125) [15]. Among these, CA-125 is the dominantly approved indicator molecule for EOC and the current clinical standard for cancer supervision. Due to the lack of specificity and low sensitivity (50%-62%), however, CA-125 levels may be elevated in other conditions such as endometriosis and other malignant tumors, including breast and lung cancers [16]. Of note, nearly 50% of ovarian neoplasm patients with normal CA-125 levels after chemotherapy showed disease persistence [17]. Thus, CA-125 has limited functionality in the context of asymptomatic women and is not recommended as a screening tool. Recently, some tumor specific DNA, i.e. circulating tumor DNA (ctDNA), was found in patient plasma, and demosntrated high correlation with ovarian cancer prognosis [18–20]. This newly non-invasive biomarker open a new page for ovarian cancer detection and diagnosis. Since the first detection of ctDNA in ovarian cancer in 2012 [21], there has been a significant progress on the role of ctDNA in ovarian cancer and detection methods, especially within the recent five years. Hence, in this review, we summarize the findings of relevant studies of this period. Furthermore, we illustrate the currently available technologies and discuss the existing challenges for analyzing ctDNA in liquid biopsy for application in ovarian cancer.

## A brief overview of ctDNA

ctDNA is emerging as one of the most promising novel alternative prognostic or diagnostic biomarkers for detecting and tracking cancer. The presence of ctDNA in the blood of cancer patients was first reported in the 1970s [22], followed by successful detection of *TP53* mutations in body fluids of patients with bladder cancer [23]. During the process of tumor apoptosis, necrosis, or active release, cell-free DNA (cfDNA) is released into the bloodstream. ctDNA is derived from a fraction of total cfDNA [24], and the half-life of ctDNA in the blood circulation is less than 2 h [25]. ctDNA consists of short DNA fragments (150–200 base pairs). This characteristic, along with its circulating half-life, makes the detection of ctDNA a promising diagnostic tool. A series of comprehensive studies encompassing multiple primary tumor types (such as ovarian, bladder, and colorectal cancers) and/or stages revealed a 6-log variation in ctDNA content [26, 27]. In addition, ctDNA has been detected in over half the cases of most cancer types [18], and has shown remarkable correlations with the molecular pathology of solid tumors [24, 28, 29]. Moreover, ctDNA may enable the visualization of the whole tumor genome rather than that of a specific section. In addition, ctDNA analysis is noninvasive for obtaining tumor tissue using biopsy and allows serial collection of samples to evaluate the quantitative and compositional changes over time. It is important that ctDNA analysis has demonstrated evolutionary adaptation in response to the inhibitors of platinum chemotherapy and poly ADP ribose polymerase (PARP). For instance, the emergence of bridging reversion mutations result from these treatments in some patients may be easily detected within ctDNA, which offer us an effective tool for cancer supervision [30–32]. Currently, with the development of the analysis and isolation of ctDNA technologies, the noninvasive testing in cancer diagnostic become more widespread [33, 34]. Taken as a whole, ctDNA is gaining momentum as a clinically feasible option capable of reflecting both spatial and temporal tumor heterogeneity (TH).

## The detection methods of ctDNA

**Fig. 1:**
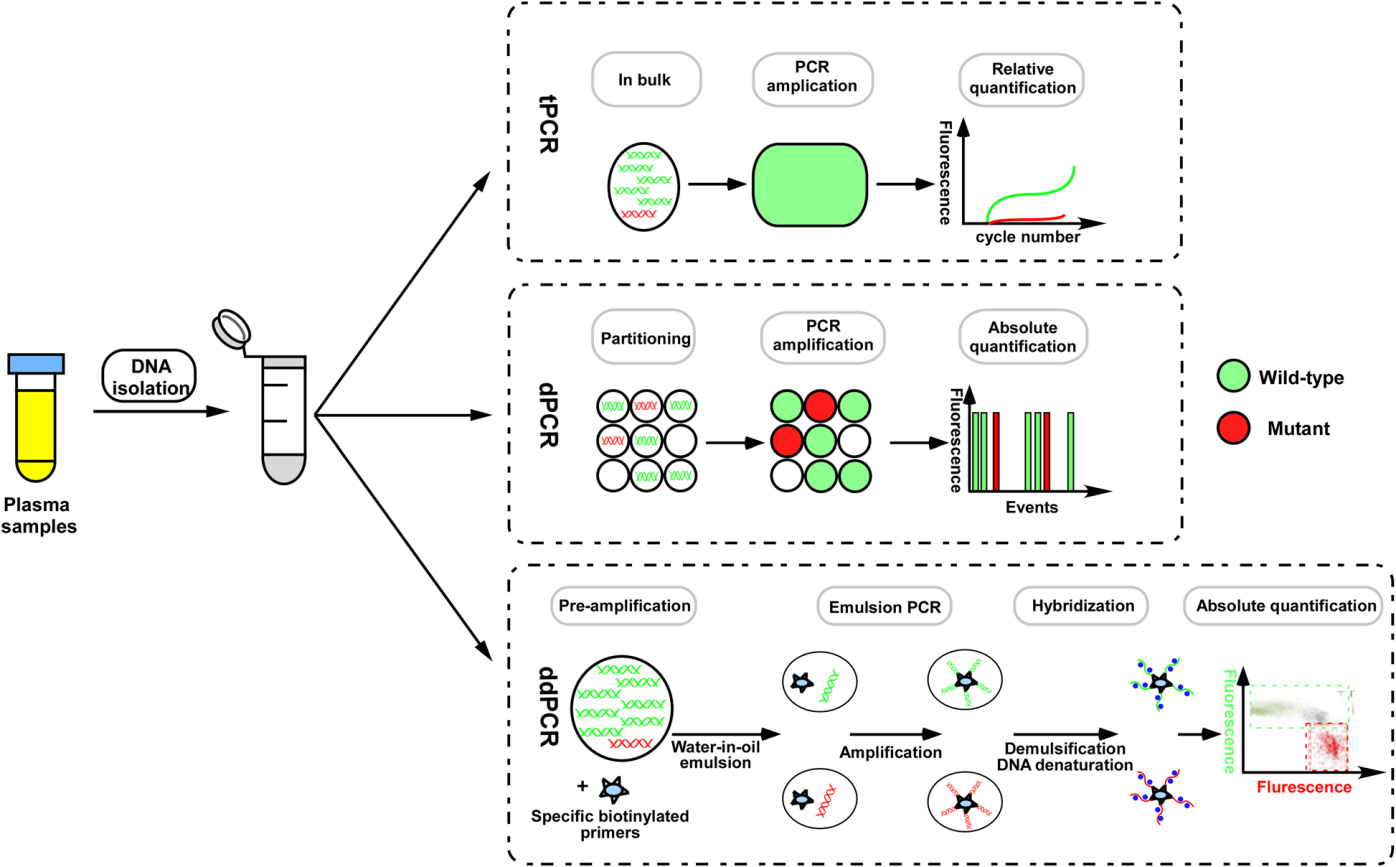
Comparison between traditional PCR (tPCR), dPCR and Droplet digital PCR (ddPCR) systems. In tPCR, mutant targets in red, and abundant wild-type sequences in green. In dPCR, the sample is partitioned into individual compartments for multiple PCR reactions in parallel and digital counting gives rise to an absolute quantification. In ddPCR, Mutant (red) and wild-type (green) fragments are both pre-amplified by multiplex PCR [49], which are performed in each droplet generating millions of identical DNA templates attached to each bead. Captured DNA fragments are denatured and hybridized with fluorescent probes specific for mutant (red) and wild-type sequences (green) [50].

In a recent prospective study, gene mutations in ovarian cancer were detected using ctDNA analysis; 94% (48/51) patients carrying mutation were identified using the blood sample which highly consistent with surgical verification and related to progression-free survival (PFS) [27]. Such high detection efficiency is attributed to the advancement of technologies in genomic analysis. Currently, multiple methods, including polymerase chain reaction (PCR)-based and next generation sequencing (NGS)-based approaches, have been developed to identify the cancer-specific mutations in the bloodstream ctDNA (NGS technologies are summarized in Table 1). PCR-based approaches have been successfully applied in ctDNA analysis; however, they are limited to detection of certain specific known mutations. In fact, a third-generation PCR technology, digital PCR (dPCR) or droplet dPCR (ddPCR), has been shown to possess a high specificity (81%) and ultra-sensitivity (99%) for a known site in ovarian cancer [26, 47, 48]. It allows absolute quantification of nucleic acids and performs target mutant or wild-type analysis of biological samples using fluorescent probes (Fig. 1). In contrast, NGS allows for highly sensitive gene detection against multiple genomic regions in a single assay and has been used for DNA mutation profiling and tumor mutation burden determination [51]. Other approaches, such as whole-genome sequencing (WGS) and cancer-personalized profiling by deep sequencing (CAPP-Seq), which use NGS for the analysis of ctDNA in ovarian cancer, have a broad range of applications, including evaluation of tumor mutation burden [52], detection of epigenetic changes, and diagnostics or identification of resistance mutations [53, 54]. Overall, ctDNA has been detected and analyzed with high diagnostic sensitivity and specificity in ovarian cancer using a variety of methods (Table 2).

**Table 1:** NGS-based methods applied in the detection of ctDNA in ovarian cancer

Technique	Targeted or nontargeted sequencing	DNA volume of plasma/blood	DNA isolation (Yes/NO)	Analytical sensitivity	Quantitative Results	Type of alterations detected	Ref.
AmpliSeq	Targeted sequencing	2 ml plasma 1-100 ng DNA	Yes	>2%	Yes	SNVs, indels	[35, 36]
Safe-SeqS	Targeted sequencing	3 ng DNA	Yes	0.1%	Yes	SNVs, indels	[37, 38]
TAm-Seq	Targeted sequencing	< 2 ml plasma	Yes	>2%	Yes	SNVs, indels	[39]
Capp-Seq	Targeted sequencing	7-32 ng DNA	Yes	0.02%	Yes	SNVs, indels	[40]
TEC-Seq	Targeted sequencing	5-250 ng of cfDNA	Yes	0.05-01%	Yes	SNVs, indels	[41]
WES	nontargeted sequencing	50 ng-1μg DNA	Yes	>1-3%	Yes	SNVs, indels; CNV, rearrangements	[42–44]
WGS	nontargeted sequencing	250 ng DNA	Yes	1%	Yes	SNVs, indels; CNV, rearrangements, chromosomal aberrations	[45, 46]

Analytical sensitivity: % mutant to wild-type abundance ratio; *Safe-SeqS* Safe-Sequencing System, *AmpliSeq* Amplicon sequencing, *TAm-Seq* Tagged-amplicon deep sequencing, *CAPP-Seq* Cancer Personalized Profiling by deep Sequencing, *TEC-Seq* Targeted error correction sequencing, *CNV* copy number variations, *SNV* single nucleotide variations, *WES* whole-exome sequencing, *WGS* whole-genome sequencing.

**Table 2:** The detection and analysis of ctDNA in ovarian cancer

Detection method	Detection rate (%)	Diagnostic Sensitivity and/Specificity	Prognostic Significance	Sample Subtype / Stage	Number of patients (>10)	Whole blood / plasma or serum	Genetic marker	Ref.
WGS	89	NR	PFS, HR=3.31, 95% CI 1.33-9.13, p=0.011	HGSOC (III-IV)	46	plasma	*BRCA1/2*	[55]
CAPP-Seq	NR	NR	NR	HGSOC III or IV (1 mucinous carcinoma III)	10	Blood (8.5 ml)	*TP53* (60%), *YAP2, SLITRK5, RET, GRM1, FAT1, LRRTM1, BRINP2, CDH9 and GRM1,* etc.	[56]
ddPCR	37.3	NR	OS (p=0.017); PFS (p<0.001)	EOCs (I-IV)	85	Plasma (0.5 ml)	*PIK3CA* or *KRAS*	[57]
MSP	70.6	Sp=50%, Sn=90.0%	NR	EOCs (I- III)	17	Plasma	CNV	[52]
Targeted-NGS	TP53=96	NR	PFS, HR=0.12 (p<0.0001)	EOCs (96% HGSOC)	97	Blood (9 ml); Plasma (2-3 ml)	*BRAC1, BRAC2, TP53*	[58]
Targeted-NGS	100 for TP53 and variable for the other genes	NR	PFS (p<0.01)	HGSOC (II-IV)	12	Blood (5-6 ml); Plasma (1-2 ml)	CNV and >500 cancer related genes including *TP53, PTEN, BRCA2,* etc.	[59]
Targeted-NGS	~90% for onlyTP53;100 for all mutant genes	Sp=100%; Sn=74-75%	OS (p=0.025); PFS (p<0.001)	EOCs (II and III)	10 drugresistant recurrent; 11 drugsensitive recurrent	Plasma (1 ml)	NV and mutant genes including *TP53, BRCA1, NOTCH2, DNMT3A,* etc.	[60]
ddPCR	10	NR	PFS (p=0.004)	Clear cell carcinoma	29	Plasma	*KRAS* and *PIK3CA*	[61]
ddPCR	93	NR	PFS, Reduced TTP (p=0.038)	HGSOC (II, III and IV)	61	Blood (15 ml); Plasma (1-5 ml)	*TP53*	[62]
WGS	16.7	NR	OS, HR=3.87 (p=0.015); PFS, HR=7.98 (p=0.045)	HGSOC (I-IV)	36	Blood (10 ml); Plasma (4 ml)	CNV	[63]
CancerSEEK	98	Sn=98%; Sp>99%; AUC=0.91 (0.90-0.92)	NR	EOCs (I-III)	54	Plasma (7.5 ml)	16 gene panel	[64]
Targeted-NGS and ddPCR	71	Sn=97.4%; Sp=100%	NR	EOCs (I-IV)	42	Plasma	55 gene panel including *TP53, KIT, ALK, APC, ERBB4,* etc.	[41]
RT-MSP	38	NR	NR	HGSOC (I-IV)	50	Blood (5 ml); Plasma (2 ml)	*ESR1*	[53]
TAm-RSeq, dPCR	BRCA1 & 2 reversion=21; TP53=79	0.031-0.085%	Reversion BRAC1/2	HGSOC (18) and endometrioid (1)(III and IV)	19	Plasma (1 ml)	*BRAC1/2*, *TP53*	[65]
TUC-BS & RRBS	33	NR	NR	HGSOC (I-IV)	151	Blood (20-40 ml); Serum (4 ml)	Regions linke to *COL23A1,C2CD4D* and *WNT6*	[66]
MSP	90	Sn=90.14% Sp=91.87%	NR	EOCs (I-IV)	149	Blood (3 ml); Serum (0.2 ml)	*OPCML, RUNX3, TFP12*	[67]
TAm-Seq, dPCR	Before treatment=82 Newly Diagnosed=86	Sn=86%	Reduced TTP, HR=0.22 (0.07-0.67; p=0.008)	HGSOC (III and IV)	40	Blood (7.5 ml); Plasma (Average = 2.1 ml)	*TP53*	[68]
WEG (WISECONDOR)	40.6	Sn=40.6%; Sp=93.8%	NR	HGSOC (I-IV)	32	Plasma	CNV	[69]
WGS	NR	Sn=67%; Sp=99.6%	NR	Invasive and borderline OC (I-IV)	57	Plasma	CNV	[70]
MN-MSP	90.1	Sn=90.14%; Sp=91.06%	NR	EOCs (I- IV)	114	Serum	*OPCML, RUNX3, TFPI2*	[71]
WES, ddPCR, TGS	93.8	Sn=81-91%; Sp: 0-99%	PFS (p=0.001); OS (p=0.0194)	HGSOC (I-IV)	22	Serum (0.2 ml)	*TP53, PTEN, PIK3CA, MET, KRAS, FBXW7, BRAF*	[47]

*NR* Not reported, *dPCR* droplet Polymerase chain reaction, *ddPCR* Droplet digital PCR, *CNV* Copy number variation, *NGS* Next generation Sequencing, *WES* Whole exome sequencing, *WGS* Whole genome sequencing, *WISECONDOR* Within-Sample copy number aberration Detector, *CAPP-Seq* cancer personalized profiling by deep sequencing, *TAm-Seq* Tagged-amplicon deep sequencing, *RT-MSP* Real-Time methylation specific PCR, *RRBS* Reduced representation bisulphite sequencing, *TUC-BS* Targeted ultra-high coverage bisulphite sequencing, *MSP* Methylation specific PCR, *MN-MSP* Multiplex nested methylated specific PCR, *PFS* Progression free survival, *OS* Overall survival, *TTP* Time to progression, *EOC* Epithelial ovarian cancer, *HGSOC* High grade serous ovarian cancer

Orthogonal validation has been performed in a limited number of studies to confirm the results obtained. Interestingly, several studies have shown that the ctDNA-based early detection and/or diagnostic assays may be further advanced by using multi-omics approaches, including those related to proteins, epigenetics, RNA, nucleosomes, exosomes, and autoantibodies [72–77]. Many of these strategies may be improved using more permissive tests with greater sensitivity, for instance, by utilizing protein biomarkers, to implement higher diagnostic effectiveness and prognostic accuracy for patients with ovarian cancer.

### ctDNA as a screening tool for tracking ovarian carcinoma

**Fig. 2:**
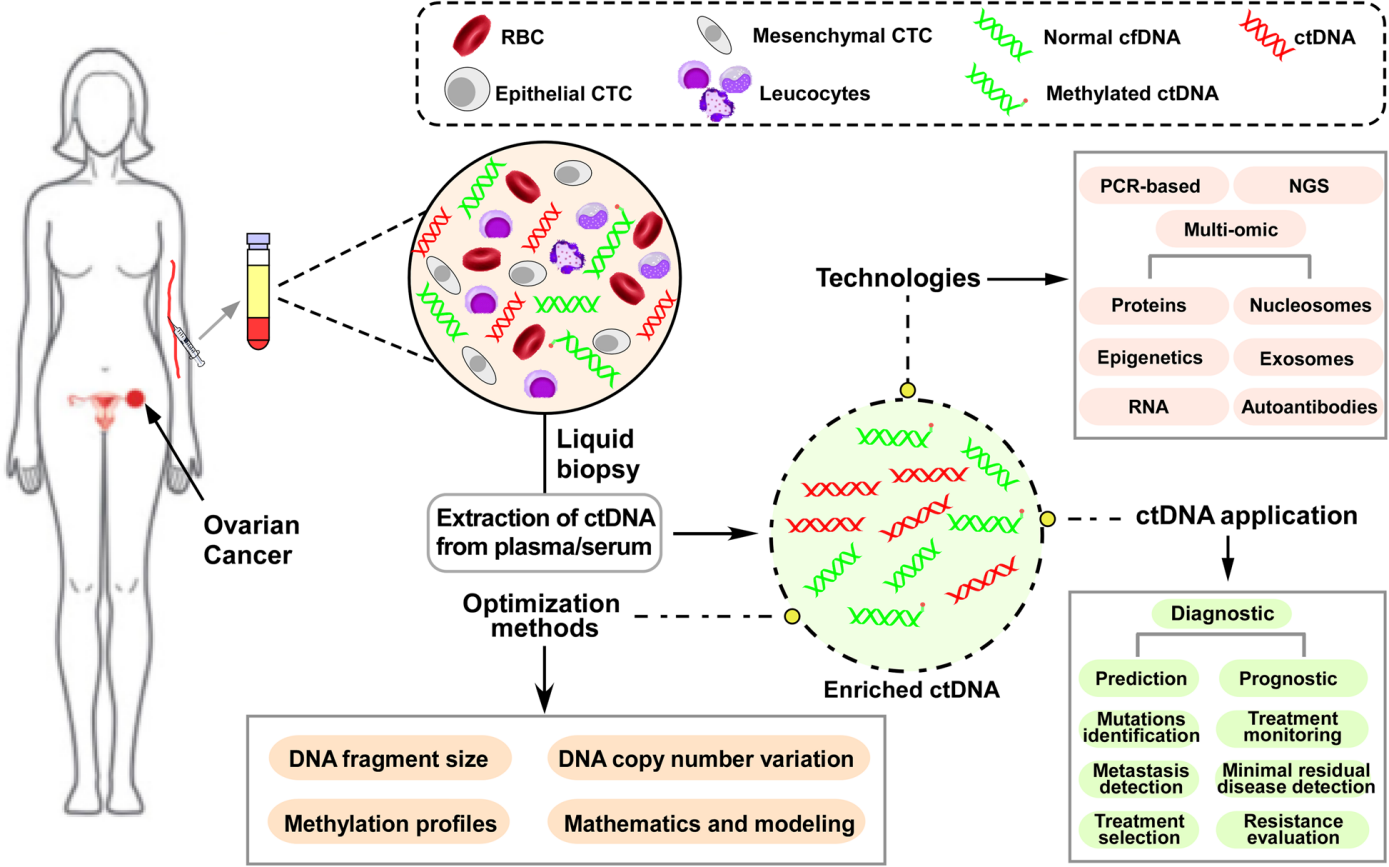
ctDNA analysis technologies, application and optimization methods in the ovarian cancer patients

As we all know, the conventional clinical and histological prognostic tools are not accurate for representing the genetic diversity of a tumor, owing to the absence of vital mutational drivers. Moreover, as ovarian cancer is a heterogeneous disease influenced by molecular evolution under treatment exposure [78], it is important to choose a suitable treatment based on the characteristics of genetic and epigenetic processes. Most of the recent developments in ctDNA testing have been directed toward using liquid biopsy. ctDNA has thus emerged as a novel promising non-invasive technology in the diagnosis, prognosis, therapy-response monitoring, resistance emergence, and clonal evaluation of patients with ovarian carcinoma and has been integrated into clinical trials to evaluate disease progression and enable direct treatment [79] (Fig. 2). For example, Phallen *et al.* [41] and Cohen *et al* [64] considered the utility of targeted error correction sequencing and the non-invasive blood test CancerSEEK technologies, respectively, to diagnose ovarian cancer and demonstrated that these methods exhibited approximately 97% sensitivity and >99% specificity.

### ctDNA is a potential biomarker for the early detection and diagnosis of ovarian carcinoma

Seventy-five percent of ovarian cancers, especially HGSOC, are diagnosed at an advanced stage, and the survival rates of EOCs have not improved significantly over the past few decades [80]. It is estimated that effective screening could reduce mortality by 10%–30%. Therefore, early diagnosis is one of the key requirements in combating this disease.

Even with the advancements in research, the development of ctDNA analysis for the early detection of EOCs demonstrates two major problems: the low abundance of tumor DNA and the high quality of background DNA in blood [81], which affect the sensitivity and reliability of diagnostic results. Thus, to analyze ctDNA in the early stages of cancer, extremely sensitive assays and risk scores for potential mutations are required. To address this point, Zhou *et al.* [82] suggested an estimated sensitivity of 70% and specificity of 90% for quantitative analysis of circulating cfDNA in ovarian cancer. These results imply that this level of specificity of ctDNA analysis is acceptable for ovarian cancer diagnosis, although extensive prospective research is needed to further assess its sensitivity, both independently and in combination with other biomarkers and methods. In addition, Widschwendter *et al.* [66] analyzed DNA fragments with high quality of background DNA in the bloodstream and demonstrated that abnormal methylation patterns of CpGs can furnish highly specific signals to indicate the presence of cancer. Meanwhile, their results indicate that the methylation patterns in ctDNA may occur prior to diagnosis in a proportion of ovarian cancers and have the potential to guide personalized therapy [66]. Moreover, NGS technology has evolved to achieve reliable sequencing of ctDNA [83], although it has not been routinely implemented in the clinic. Rothwell *et al*. [84] showed that the ctDNA data obtained by tumor characterisation to guide experimental targeted therapy (TARGET) were in concordance with the matched tumor. Actionable mutations were identified in 41 (out of 100) patients in the study of whom received a corresponding therapy. Cohen *et al*. [85] reported that by developing CancerSEEK, they were able to achieve early detection rates of 69% or better in ovarian carcinoma. These results demonstrated that the use of ctDNA could support the selection of patients for early-phase clinical trials.

### ctDNA in identification of mutations and characterization of TH in ovarian cancer

ctDNA extracted from plasma during the course of tumor apoptosis, necrosis, or active release has the potential for transformative applications in ovarian cancer. It is important that ctDNA exists as 150-200 base pairs short fragments that are able to perform PCR-based and NGS-based analyses, thereby probing the mono-mutations within cancer using allele-specific assays. Ogasawara *et al.* [57] obtained approximately 30% mutation rates of targeted ctDNA (i.e., *KRAS* or *PIK3CA*) from the patients with an ovarian tumor using ddPCR. Of note, Forshew *et al .*[21] identified mutations in the tumor suppressor gene *TP53* using Tam-Seq analysis of ctDNA from plasma samples of 46 patients with advanced ovarian cancer and achieved a sensitivity and specificity of >90%. Beside the detection of instinct mutation of the tumor, ctDNA reflects the dynamic state of targeted gene mutations. For example, two research groups detected reversion mutations of *BRCA1/2* in ovarian cancer in the process of resistant to the platinum or PARP inhibitors [32, 58]. These results indicate that this low-cost, high-throughput method may not only facilitate the analysis of ctDNA through a noninvasive “liquid biopsy” for personalized cancer diagnosis but also capture the escape mutations.

Additionally, TH is another potential issue associated with the treatment of ovarian cancers. Very few research currently have investigated the application of ctDNA analysis in addressing TH. Noguchi *et al.* [27] recently elucidated the possibility of using ctDNA analysis for supervising treatment responses, including “tumor evolution”, which reflects the evolutionary changes in TH in the N-acetylcysteine-treated patients with advanced ovarian cancer [86]. In addition, Paracchini *et al*. [55] showed shallow WGS as an inexpensive and useful tool to monitor TH in HGSOC and to obtain an accurate “snapshot” of the tumor genome compared to CA-125, the routine serum biomarker.

### Comprehensive profiles of ctDNA to response to treatment in ovarian cancer

Traditional treatment of ovarian cancer relies on imaging and surgical biopsy. In advanced or recurrent ovarian cancer patients, the former approach only determines the size of the tumor and the extent of changes, and the latter is invasive and sometimes difficult to obtain standard samples. It is well documented that the half-life of the blood protein biomarker CA-125 ranges from 9 to 44 days with the limitations of responding to biological therapies [87–89], implying the seminal advancements in the treatment of ovarian cancer need to continuously explore. A variety of ctDNA quantification methods have several advantages for assessing tumor burden to cure primary and metastatic diseases [90, 91]. The ctDNA extracted from peripheral blood samples can provide a contemporaneous profile of the tumor genomic landscape. Importantly, ctDNA not only correlates with an early foundation and identification of ovarian cancer but can also be used to monitor treatment efficacy and determine the best treatment method to improve the therapeutic effect [72]. For example, Kim *et al*. [62] reported that *TP53*-mut ctDNA demonstrates potential as a tumor-specific biomarker for monitoring treatment response in HGSOC, which is more sensitive than CA-125. Furthermore, a study from Arend *et al.* group [92] showed that the *TP53* variants in ctDNA following neoadjuvant chemotherapy were associated with the treatment response in ovarian cancer patients. Of note, Martignetti *et al*. [93] identified a rare tumor-specific fibroblast growth factor receptor 2 (*FGFR2*) fusion gene to monitor the treatment efficacy of advanced ovarian cancer using PCR-based ctDNA, and demonstrated that only the tumor cells with *FGFR2* fusion gene ctDNA derived from the patient were sensitive to FGFR2 inhibitor (BGJ398), compared to the cells from other patients. Besides, Noguchi *et al.* [27] recently reported a series of cancer-specific gene mutations identified via ctDNA from the plasma of 51 pre-treatment patients at different stages (I–IV) of ovarian carcinoma. 48 of 51 (94%) patients were found one or more non-synonymous mutations, including HGSOC (*TP53*, 66.7%), clear cell carcinoma (*APC*, 30.8%), endometrioid carcinoma (*PIK3CA*, 40%), and mucinous carcinoma (*KRAS*, 66.7%). It is worth noting that patients with any such pathogenic mutations demonstrated markedly low PFS (*p* = 0.048). Overall, the evidence on clinical utility of ctDNA may lead to personalized therapeutic strategies, and guide the clinicians to develop new therapeutic methods to avoid non-effective treatments and improve the outcome of patients.

### Prognostication and detection of ovarian minimal residual tumor

Many tumor patients that undergo curative surgery of their tumor experience recurrence, usually at distant metastatic sites seeded from the primary tumor, and present several mutations. After a surgical resection, it is difficult to distinguish between patients actually achieving remission from those having minimal residual disease. A few studies have demonstrated the ctDNA analysis can prognosticate disease recurrence and minimal residual tumor and may predict progression or response to treatment more rapidly than imaging or CA-125 [57, 94, 95]. Pereira *et al.* [94] indicated that the utility of personalized ctDNA can identify the presence of residual tumor. They also showed that the predictive lead time associated with the detection of ctDNA is 7 months over that of CT scans. In an exploratory analysis using ctDNA as a biomarker to assess treatment response in ovarian minimal residual tumor, Parkinson *et al*. [95] demonstrated that ctDNA in patients with relapsed HGSOC correlates with the tumor size at the start of the treatment. They also highlighted that patients with ctDNA levels exhibiting a >60% decrease have a remarkably longer time of progression than those with ctDNA levels decreased by 60% or less after a course of treatment. Arend *et al.* [92] recently reported that the *TP53* variants in ctDNA after neoadjuvant chemotherapy may contribute in determining the presence of minimal residual disease. Additionally, Harris *et al.* [87] identified plasma ctDNA that permitted the monitoring of cancer patients for relapse and improved therapeutic efficacy via somatic chromosomal rearrangements. Recently, ctDNA from plasma was discovered to be an independent factor for overall survival (*p =* 0.025) and PFS (*p =* 0.001) in patients with recurrent ovarian cancer [60]. These evidence suggest that ctDNA levels are correlated with the recurrence of cancer in patients and highlight the possibility of utilizing the detection of ctDNA mutations as an early indicator of recurrence.

## Optimizing ctDNA detection

The clinical utility of ctDNA in ovarian carcinoma as a diagnostic biomarker has gradually advanced as a minimally-invasive and real-time surrogate for visualizing the development of tumor. However, the advancement in this field of research is relatively recent. In fact, the development of ctDNA analyses was initially impeded by a lack of specific and sensitive techniques for cfDNA quantification. The recent dPCR- or NGS-based assays have advanced critical parameters, such as the mutation allele fraction (MAF) or variant allele fraction (VAF) for the analysis of ctDNA. A large number of ctDNA molecules are expected to possess these two parameters. For example, while the MAF has been reported to be less than 0.1% in a metastatic setting [39, 96], it enables the detection of ctDNA. However, there are two pre-analytical problems associated with optimal ctDNA analysis, including a technical and a biological issue. The technical problem is that many non-designated “mutations” are generated during the progress of analysis. Even in high-throughput screening, the error rate of the compound is higher than that of the available methods. The biological problem is that even with substantial advancement of technologies, the actual samples from plasma or bloodstream (about 5–10 ml) may limit the ctDNA mutation analysis. Hence, the optimization of such technical and biological limitations might lead to new avenues for the clinical applications of this technology.

### Potential challenges in the application of ctDNA analysis

Currently, several potential obstacles exist in the development of a ctDNA-based early tumor diagnostic tool. As noted above, an early detection of tumor is critical in the treatment or morbidity. Therefore, the identification of cancer stage without any prior knowledge of the cancer-specific mutations is challenging and may not be effective in assessing all cancer-associated genes. The cost of ctDNA analysis methods (i.e. NGS) is likely to decrease with further development of the technology. However, even with a reduced cost, the current methods might limit the detection to specific genes or portions of genes, rather than enabling the evaluation of all known genes associated with cancer. Another challenge for early detection is the fact that the cancer may not shed adequate amounts of ctDNA in early-stage disease or during micrometastasis due to the lower disease burden. Therefore, to promote the clinical application of ctDNA analyses, novel approaches for sample collection may be required in the future to stabilize blood cells and/or reduce the contamination of background DNA in the serum or plasma samples. The third foreseeable hurdle is clonal hematopoiesis, which has not been identified in the affected tissue or organ in an occult disease, because of complex ctDNA mutations [97]. Finally, the detection of multiple mutations could offer invaluable information regarding the origin of cancer in a single patient but is not feasible since the infrequency or specificity of ctDNA. At the same time, the mutant ctDNA may display significant false positivity due to non-cancer derived mutations.

### Optimization methods

Although the development of ctDNA analysis is decelerated by some barriers against its clinical utility, certain natural features of ctDNA may strengthen its ability to serve as a diagnostic tool. Plasma cfDNA originating from a neoplasm is considered as ctDNA, and its levels are higher in cancer patients compared to those in healthy individuals [98]. Most studies have mainly focused on genomic alterations for monitoring the tumor and have ignored the changes in ctDNA fragment lengths. Mouliere *et al.* [99] noted that the size of the DNA fragments (90–150 bp) could be optimized to increase the sensitivity of ctDNA detection. As a result, the median enrichment of ctDNA improved over two-fold in >95% of cases. Cristiano *et al*. [100] further determined that fragmentation profiles may be used to directly detect ctDNA during the early stages of ovarian carcinoma. Another intrinsic feature of ctDNA is the copy number variation (CNV). Because of the small ctDNA fraction derived from early-stage tumors (<1%), CNV presents a hurdle in attaining high-sensitivity testing based solely on a mutational assay. Molparia *et al.* [101] suggested the potential of CNV detection for ctDNA-based screening as a broad screening methodology. In addition, the CancerSEEK study combined the analysis of specific mutations in ctDNA with a machine-learning algorithm to achieve an accurate diagnosis of ovarian cancer at a sensitivity of 98% and specificity of >99% [64]. Analysis of clonal hematopoiesis mutations in blood with algorithm may also improve the specificity for mutant DNA analysis in ovarian cancer treatment studies. Finally, the methylation profiles may be employed to investigate a possible association between methylation rate of cancer-specific genes and ctDNA [81, 102]. Such methylation patterns in ctDNA can be used to monitor the development of ovarian cancer [52]. Recently, certain mathematical models of ctDNA have been designed for the prediction of tumor volume and to enhance its utility as a tool for monitoring tumor progression. For example, McPherson *et al.* [103] and Fiala and Diamandis [29] showed that a higher concentration of ctDNA is related to advanced cancer, as well as metastatic cell growth patterns. In addition to ctDNA in blood, ctDNA from ascites correlates with DNA mutations present in tumor biopsies of ovarian cancer. Accordingly, the analysis of fragment size, CNV-based screening, mathematical algorithm models, and ctDNA-associated methylation can facilitate the detection of ctDNA and may provide an alternative approach to the prediction of cancer progression.

## Future challenges and prospective

As described above, ctDNA provides an extremely promising treatment strategy as a noninvasive tool for monitoring ovarian carcinoma during diagnosis and prognosis and while tracking evaluations of patients. In contrast to other traditional biomarkers, such as CA-125, ctDNA demonstrates substantial potential as a key component of ovarian cancer detection trials. Similarly, ctDNA can potentially monitor minimal residual tumor and disease recurrence. Although ctDNA displays a broad prospect in ovarian cancer monitoring, there are several key points that need to be addressed as a priority in the future. First, increase in the accuracy and prevention of the error of ctDNA detection originating from the measurement technique is a critical issue, since a reliable result is key for evaluating the progress of ovarian cancer. Moreover, most ctDNA biomarkers of ovarian cancer currently used in the clinical tests usually detected in other types of cancers as well, leading to possible confusion and interfering with accurate judgment of ctDNA in ovarian cancer supervision, especially in its prediction. Thus, discovering specific ctDNAs originating from ovarian cancer is necessary to improve the detection rate in early screening and to reduce the incidence of this cancer type. Additionally, although several improvement methods mentioned above are being currently employed to optimize the application of ctDNA in ovarian cancer detection, there is a vast scope for advancement. The development of novel methods or combining different high-throughput sequencing and other clinical testing methods to unravel the inherent characteristics of ctDNA is impending and can provide novel insights for into ctDNA-based ovarian cancer detection approaches. More importantly, since ovarian cancer is a heterogeneous disease and ctDNA content can be affected by the tumor stage, heterogeneity, and clonality, the relevance of ctDNA in the clinical treatment of different types, stages, or sizes of ovarian cancer needs to be established in order to provide a clear guidance for cancer monitoring and treatment, which warrant further studies in clinical trials with a significant number of cases. In conclusion, ctDNA acts as a unique biomarker in ovarian cancer management and goes beyond the utilization and assessment of known tumor-specific mutations. Thus, further research is required to establish its clinical utility and ultimately improve personalized or precise treatment of the patients.

## Data Availability

Not applicable

## Abbreviations

CA-125: Cancer antigen 125
CNY: Copy number variation (CNV)
ctDNA: Circulating tumor DNA
cfDNA: Cell-free DNA
CSCs: Cancer stem cells
dPCR: Digital PCR
ddPCR: Droplet dPCR
EOC: Epithelial ovarian cancer
FGFR2: Fibroblast growth factor receptor 2
HGSOC: High-grade serous ovarian cancer
MAF: Mutation allele fraction
NGS: Next generation sequencing
PARP: Poly ADP ribose polymerase
PCR: Polymerase chain reaction
PFS: Progression-free survival
sWGS: Shallow whole genome sequencing
TARGET: Tumor acARacterisation to Guide Experimental Targeted therapy
TFR1: Transferrin receptor 1
TH: Tumor heterogeneity
VAF: Variant allele fraction
WGS: Whole genome sequencing
